# Neurological status in paediatric upper limb injuries in the emergency department – current practice

**DOI:** 10.1186/1756-0500-5-324

**Published:** 2012-06-22

**Authors:** James S Robertson, Andrew G Marsh, James S Huntley

**Affiliations:** 1 Orthopaedic Department, Royal Hospital for Sick Children, University Glasgow, Yorkhill, Glasgow, G3 8SJ, UK

**Keywords:** Trauma, Paediatric Injury, Paediatric Emergency Medicine, Clinical Assessment, Musculoskeletal

## Abstract

**Background:**

In upper limb injuries it is important to assess associated neurological injury. The aim of this study was to assess the initial (Emergency Department (ED)) documentation of neurological status in paediatric patients presenting with upper limb injuries.

**Findings:**

Case notes of paediatric patients admitted to the orthopaedic ward with upper limb injuries were retrospectively collected over a three month period. Initial ED documentation was recorded and case notes examined for any neurological deficit on admission. Of the 121 patients, 107 (88.4%) of case notes had some form of neurological documentation. The remaining case notes (*n* = 14, 11.6%) had no mention of neurological examination. There were 10 (8.2%) patients with pre-operative neurological deficits identified; none of these had been previously identified by the ED.

**Conclusion:**

There are failings of neurological documentation on the part of ED staff. It is likely that these reflect a knowledge deficit in the examination of the injured upper limb in paediatric patients.

## Findings

Approximately 25% of childhood injuries are fractures with 82.2% involving the upper limb [1,2]. Neurological deficit occurs in 1% of paediatric forearm fractures, increasing to 14% for open injuries [3,4]. In addition, distal humeral supracondylar fractures are associated with neurological deficit in up to 20%. This most commonly involves the anterior interosseous nerve (AIN) although radial, median and ulnar nerves can all be involved [5,6].

Many upper limb fractures require manipulation or operative intervention. At our centre in 2008, 200 out of 314 (64%) non-torus forearm fractures required treatment under general anaesthetic [7]. Neurological assessment is important to determine associated nerve injury at presentation. The documentation of neurological status prior to theatre is important so that any subsequent iatrogenic nerve injury can be diagnosed.

Neurological assessment should involve a thorough motor, sensory and vascular examination. In paediatric patients neurological assessment can be challenging due to difficulties with patient understanding and compliance.

The aim of this project was to assess quality of documentation of neurological assessment in children presenting with upper limb fractures to the emergency department.

## Methods

The clinical notes of all children admitted into the orthopaedic unit from the emergency department with upper limb injuries were retrospectively reviewed over three months (May 2011-July 2011). The inclusion criteria were that the child had sustained an upper limb injury requiring admission for orthopaedic intervention under general anaesthetic. Patients were identified through ward records. 124 patients were identified over the three month period; of these 121 were obtained (the remaining three initial ED assessments were untraceable). Patient demographics, injury sustained and mechanism, initial treating hospital and definitive management were recorded.

Ethics committee approval was not required as this was a simple medical audit with no intervention, clinical or otherwise.

Documentation of neurological assessment performed in the emergency department was noted and compared to the examination findings of the admitting orthopaedic surgeon.

## Results

In this three month period, there were 121 admissions (61 male; 60 female) to the orthopaedic ward for an upper limb injury requiring intervention under general anaesthetic. The age range was from 1 year old to 12 years. Table 1 shows the distribution of patient ages. There were eight different injury categories (Table 2). The commonest injury was a combined radius and ulnar fracture (*n* = 48, 39.8%). The mechanisms of injury varied with the most common injury being a simple fall (*n* = 51, 42.1%). Other mechanisms were: monkey bars/climbing frame (*n* = 20,16.5%), trampoline (*n* = 20, 16.5%), sport (*n* = 11, 9.1%), swings (*n* = 9, 7.4%), bike (*n* = 8, 6.6%) and slides (*n* = 2, 1.6%).

**Table 1:** Distribution in age of patients admitted to the orthopaedic ward

**Age**	**Number**	**%**
0-2 Years	8	6.6%
3-5 Years	36	29.8%
6-8 Years	41	33.8%
9-12 Years	36	29.8%

**Table 2:** Distribution of injuries amongst patients (all ages/aged 3 or over)

**Type of Injury**	**All Ages (121)**	**Age 3+ (113)**
Radius & Ulna #	48 (39.6%)	45 (39.8%)
Radius #	33 (27.3%)	32 (26.4%)
Supracondylar #	22 (18.2%)	19 (16.8%)
Lateral Condyle #	8 (6.6%)	7 (6.1%)
Medial Condyle #	3 (2.5%)	3 (2.7%)
Dislocated Elbow	3 (2.5%)	3 (2.7%)
Ulna #	3 (2.5%)	3 (2.7%)
Olecranon #	1 (0.8%)	1 (0.9%)

As the hospital is a tertiary referral centre, there were children presenting from several different emergency departments throughout the country. Sixty (49.6%) patients presented initially to the base hospital and 61 (50.4%) of patients were seen initially in the emergency departments of other hospitals.

An attempt at a form of neurological status documentation was made in 107 cases (88.4%). There was no mention of a neurological examination or neurological status in 14 (11.6%). Documentation involved the following: terms “NVI” (*n* = 72, 59.5%), “CSM” (*n* = 30, 24.8%), “Sensation” (*n* = 3, 2.5%) and “Moving” (*n* = 2, 1.7%).

In 114 case notes (94.2%) there was no documentation of particular nerves being examined. The anterior interosseous nerve (AIN) was never mentioned.

During the three month period there were 10 patients with neurological deficits (8.2%). These were found in a variety of injuries (Table 3). However, none of these deficits were picked up by initial examination in the ED. The most common injury producing a neurological deficit was a supracondylar fracture (*n* = 4, 40%). Of the nineteen supracondylar fractures seen in children over the age of three, there were four neurological deficits (*n* = 4, 21%), none of which were identified on initial examination.

**Table 3:** Neurological deficit discovered, the injury sustained and the initial examination in the emergency department

**Age**	**Neurological Deficit**	**Injury**	**Initial Documentation**	**Hospital Attended**
12	Ulna Paraesthesia	Dislocated Elbow	Nil	Other
8	Median Paraesthesia	Ulna & Radius #	CSM	Other
8	Median Paraesthesia	Radial #	NVI	Base
8	Radial Neurapraxia	Supracondylar #	NVI	Base
8	AIN Palsy + Median Paraesthesia	Supracondylar #	NVI	Base
7	Median Paraesthesia	Supracondylar #	NVI	Other
8	Median Paraethesia	Radial #	NVI	Base
9	Radial Palsy	Ulna & Radius #	Movement	Base
11	Ulna Paraesthesia	Ulna & Radius #	CSM	Base
6	AIN Palsy	Supracondylar#	Nil	Other

## Discussion

When assessing upper limb injuries, neurological examination and clear documentation is essential. Here, we have shown that neurological examination in paediatric upper limb injuries presenting to the emergency department are incompletely documented and significant nerve injuries are missed. Over 90% of cases in this study had no documentation of individual nerve examination (i.e. which upper limb nerves were assessed and what motor or sensory examination had been performed). This included all 10 cases which had an associated neurological injury.

Neurological examination can be challenging in paediatric patients where communication and understanding of specific movements can be limited. Some emergency departments may not routinely assess paediatric injuries. In addition, ED staffing is also subject to frequent change with more junior staff (e.g. FY2s) on shorter rotational programmes. Therefore these doctors may have little experience of paediatric injury assessment.

Every child with an upper limb injury should routinely undergo examination of the radial, ulnar, median and AIN. A simple, structured approach to examination should be used which is easy for a child to understand and obey. Davidson (2003) reported a simple method of examining gross neurological function of the hand [8]. He describes the childrens’ game, rock-paper-scissors to assess motor function of the median, radial and ulnar nerves. The median nerve flexes the wrist and fingers into a fist for rock. The radial is tested by extending the fingers and metacarpophalangeal joints to produce paper. The scissors are created by clawing the ring and little finger and abducting the extended index and middle finger [8]. A simple way to assess the AIN has also been developed by producing an ‘OK’ sign with your fingers. This tests the motor power to your flexor pollicus longus and the deep flexor of the index finger as described classically by Spinner (1970) [9]. Performing the simple tasks of “rock-paper-scissors-ok” may prove a useful initial screening tool for picking up neurological deficits in the ED.

A limitation of our study is that neurological deficit may occur following the initial ED assessment. It is therefore possible - though we would argue unlikely - that in some of our 10 patients with neurological deficits, the initial examination was carried out accurately with the deficit developing later. We are seeking to address this question with a study of the ED’s knowledge of neurological examination and potential deficits.

It is essential that nerve injuries at presentation are differentiated from iatrogenic neurological deficits. A missed neurological deficit can have medical and medico legal complications. Iatrogenic neurapraxias account for around 2–3% of nerve injuries in paediatric supracondylar fractures [6]. The median nerve can become incarcerated in the fracture following reduction and ulnar nerve injuries are not infrequent after percutaneous fixation [10].

In conclusion, our study shows that although neurological examination of paediatric patients presenting with upper limb fractures is usually performed in the ED, it tends to be incompletely documented and neurological injuries may be missed. A simple rock-paper-scissor-ok guideline may be of benefit.

## Competing interests

The authors declare that they have no competing interests.

## Authors’ contributions

AGM conceived of the idea for the project. All authors contributed to the design of the project; JSR collated the data and wrote the first draft. All authors revised/rewrote the manuscript and discussed this at revisional stages. All authors read and approved the final manuscript. JSH is the guarantor.
